# Age-specific association between meal-skipping patterns and the risk of hyperglycemia in Korean adults: a national cross-sectional study using the KNHANES data

**DOI:** 10.1186/s12889-024-18762-w

**Published:** 2024-06-25

**Authors:** Yeonji Kang, Minji Kang, Hyunjung Lim

**Affiliations:** 1 Department of Medical Nutrition, Graduate School of East-West Medical Science, Kyung Hee University, Yongin, 17104 Republic of Korea; 2 Research Institute of Medical Nutrition, Kyung Hee University, Seoul, 02447 Republic of Korea

**Keywords:** Meals, Intermittent fasting, Hyperglycemia, Prediabetic state, Diabetes Mellitus, type 2, Circadian clocks, Republic of Korea

## Abstract

**Background:**

Glucose metabolism regulation is influenced by age and meal skipping, although research on their interplay with hyperglycemia remains limited. This study aims to explore the intricate relationship between meal-skipping patterns and hyperglycemia risk across distinct age groups in South Korean adults.

**Methods:**

Utilizing data from the Korea National Health and Nutrition Examination Surveys (KNHANES) conducted from 2013 to 2020, comprising 28,530 individuals aged 19 years and older, this study employed multivariable logistic regression models to examine the associations between meal-skipping patterns and the risk of hyperglycemia.

**Results:**

Meal-skipping patterns were categorized into three groups: no skipping (NS), skipping breakfast (SB), and skipping dinner (SD). Age groups were defined as “young” (aged 19–44), “middle-aged” (aged 45–64), and “elderly” adults (over 65 years old). Among “young” adults, SB was associated with a 1.33-fold higher risk of hyperglycemia (OR = 1.33, 95% CI = 1.14–1.54) compared to NS. Conversely, in “elderly” adults, SD was linked to a 0.49-fold reduced risk (95% CI = 0.29–0.82) when compared to NS. Additionally, we observed that the Korean Health Eating Index (KHEI) scores, representing the quality of diet on a scale of 0 to 100, were consistently lower in SB compared to NS across all age groups. Intriguingly, specifically among the “elderly” group, this score was higher in SD compared to NS (*p* < 0.001).

**Conclusions:**

This study demonstrates age-specific variations in the association between meal-skipping patterns and the risk of hyperglycemia.

**Supplementary Information:**

The online version contains supplementary material available at 10.1186/s12889-024-18762-w.

## Introduction

In contrast to historical dietary guidelines primarily focused on food choices [1–3], contemporary dietary trends, exemplified by intermittent fasting, underscore the significance of meal timing [4]. Intermittent fasting, characterized by cycles of fasting for varying durations (e.g., 12 h daily or 1 to 2 meals a day), has garnered attention for its potential health benefits. Accumulating research suggests intermittent fasting may enhance physical function, increase insulin sensitivity, extend lifespan, facilitate weight loss, reduce systolic and diastolic blood pressure, and fortify the immune system [5–7]. Consequently, an increasing number of individuals in South Korea intentionally skip meals for various health benefits.

However, several studies [8–10] have linked meal-skipping, especially breakfast omission, to an elevated risk of developing type 2 diabetes. This relationship can be attributed to the abnormal insulin secretion due to circadian clock disruption [11]. Hyperglycemia can result from decreased insulin secretion, diminished glucose utilization, and increased glucose production [12], underscoring the intimate connection between hyperglycemia and glucose metabolism [13]. The circadian clock orchestrates glucose metabolism by regulating the activities of metabolic enzymes, hormones, and transport systems [14]. Meal-skipping disrupts this circadian rhythm, with various studies [15, 16] associating breakfast omission and late-night eating with elevated levels of glycated hemoglobin and postprandial hyperglycemia, highlighting the importance of regular meal consumption for individuals with diabetes. However, most studies on meal- skipping have primarily focused on breakfast, making it challenging to establish links with other forms of meal omission. Furthermore, age plays a pivotal role in abnormal glucose metabolism [17], yet many studies have either treated age as a covariate or concentrated on specific age groups, limiting cross-age group comparisons. Lastly, there has been a scarcity of research conducted exclusively on Korean populations.

This study classified Korean adults into there age groups: young, middle-aged, and elderly, and examined the relationship between meal skipping patterns and hyperglycemia in each age group.

## Research design and methods

### Data source

This study utilized data sourced from the Korea National Health and Nutrition Examination Surveys (KNHANES), spanning the years 2013–2015 (KNHANES VI), 2016–2018 (KNHANES VII), and 2019–2020 (KNHANES VIII-1.2). KNHANES has been conducted continuously since 2007, employing a rolling survey sampling method conducted throughout the year. This comprehensive survey comprises a health interview, health examination, and nutrition survey. Information pertaining to sociodemographic characteristics was extracted from the health interview data. Measurements such as anthropometry, blood pressure readings, and laboratory tests for the participants were conducted during direct health examinations at a mobile examination center. The response rates for each iteration of KNHANES were as follows: 78.3% for KNHANES VI, 76.6% for KNHANES VII, 74.7% for KNHANES VIII-1, and 74.0% for KNHANES VIII-2, respectively. Detailed descriptions of the study’s design and data collection procedures have been previously published [18].

### Study population

This study focused on adults aged 19 and older, resulting in an initial sample of 50,162 individuals possessing comprehensive demographic data. We meticulously excluded 8,505 participants whose meal-skipping patterns could not be reliably discerned and 7,446 participants who reported implausible energy intake levels (< 500 or > 5000 kcal per day) [19]. Furthermore, 1,602 pregnant women were excluded due to the hormonal fluctuations associated with pregnancy. To ensure a meaningful analysis, we excluded 4,079 individuals for whom hyperglycemia classification was not possible due to the absence of blood sugar indicators or a doctor’s diagnosis. Consequently, our final study cohort comprised 28,530 adults (13,073 men and 15,457 women). Within this refined sample, participants were systematically categorized into three distinct age groups: the “Young” Group (aged 19–44), consisting of 9,013 individuals. The “Middle-Aged” Group (aged 45–64) encompasses 11,168 individuals, and the “Elderly” Group (aged 65 or older) comprises 8,349 individuals.

### Dietary assessment

The KNHANES incorporates a comprehensive array of nutrition survey components, encompassing an eating habits survey, a food frequency questionnaire (FFQ), a 24-hour recall (24RC), a food safety survey, and a Korean Health Eating Index (KHEI). The eating habits survey is designed to elicit information about participants’ typical eating habits and nutritional knowledge. Meanwhile, the FFQ evaluates the frequency of food consumption over the preceding year. The 24RC provides detailed data on the types and quantities of foods consumed on the day immediately preceding the survey, offering valuable insights into participants’ dietary intake. Additionally, the food safety survey assesses various aspects of dietary circumstances. The KHEI, a well-established tool in dietary evaluation [20], is employed in this study to assess participants’ habitual diets. It assigns a score out of 100 based on 14 distinct categories, with higher scores indicative of superior diet quality. In our investigation, meal-skipping patterns were classified utilizing responses from the eating habits survey, while dietary assessments for each pattern were conducted through the integration of 24RC and KHEI data.

### Anthropometric and biochemical measurements

Using standardized techniques and equipment, height and weight were measured with subjects in light clothing and without shoes. Waist circumference was measured at the midpoint between the upper iliac crest and the lowest rib. Blood pressure was measured three times in a seated position after a 10-minute rest. All blood samples were collected after an 8-hour fast in the morning, and biochemical levels, including FBG, were determined using an automated blood analyzer (Hitachi 7600, Hitachi Instruments Inc, Japan) in a certified central laboratory.

### Classification of meal-skipping patterns

In this study, we defined each meal-skipping pattern based on participants’ weekly meal frequency for breakfast, lunch, and dinner over the past year, as reported in the dietary habits survey of the KNHANES nutrition survey. Participants who reported eating a specific meal 5–7 times a week were categorized as ‘eating’ that meal, while those who reported eating it 0–2 times a week were categorized as ‘skipping’ it. Responses indicating 3–4 meals per week were considered ambiguous and excluded from the analysis due to small number of subjects.

Participants were subsequently divided into three groups based on their meal-skipping patterns:

- No skipping (NS): This group included individuals who consumed breakfast, lunch, and dinner five or more times a week.
- Skipping Breakfast (SB): Participants in this category had breakfast less than twice a week.
- Skipping Dinner (SD): This group consisted of subjects who had dinner less than twice a week.

The SB and SD groups experience a fasting period of over 15 hours when skipping either breakfast or dinner, whereas the SL group, which skips lunch, has a fasting period of approximately 10 hours. The ‘Skipping Lunch (SL)’ group, consisting of 927 participants with short meal intervals, did not show statistically significant results across all age groups. Consequently, this paper does not present it as a conclusive meal-skipping pattern analysis result. The analysis included 28,530 subjects, with 21,314 in the NS group, 6,836 in the SB group, and 380 in the SD group.

### Classification of hyperglycemia

Following the criteria established by the American Diabetes Association [21] and the Korean Diabetes Association [22], the diagnosis of diabetes was based on the presence of any of the following conditions: fasting blood glucose (FBG) levels of 126 mg/dL or higher, glycated hemoglobin (HbA1c) levels of 6.5% or greater, current use of diabetes medication or insulin injections, or a documented doctor’s diagnosis of diabetes. Additionally, pre-diabetes was identified when FBG levels fell within the range of 100 mg/dL to 125 mg/dL or HbA1c levels ranged from 5.7 to 6.4%. In this study, we operationalized hyperglycemia as the collective occurrence of either diabetes or pre-diabetes within the study population.

### Statistical analysis

In the KNHANES, a complex sampling design was employed to enhance the representativeness of the sample and the accuracy of the estimates. To ensure a credible analysis reflecting the entire population of the Republic of Korea, we utilized the final weights recommended by the Korea Centers for Disease Control and Prevention during our analysis. Statistical analyses were carried out using SAS version 9.4, accounting for the intricate sampling design through stratified and cluster variables as well as weights. Categorical variables were assessed with chi-square tests, and continuous variables were analyzed using ANCOVA. Multivariable logistic regression models were used to estimate odds ratios (OR) and 95% confidence intervals (CI) for associations between meal-skipping patterns and hyperglycemia risk (prediabetes and type 2 diabetes). Four models were applied progressively: unadjusted (Model 1), adjusted for sex and energy intake (Model 2), further adjusted for general characteristics (marital status, household income, graduation status, physical activity, residential area, smoking, and drinking) (Model 3), and augmented with Body Mass Index (BMI), Waist Circumference (WC), and family history (Model 4). Significance was set at *p* < 0.05. This approach ensured a comprehensive examination of meal-skipping pattern associations with hyperglycemia risk while considering potential confounders.

## Results

### Socioeconomic demographics and health-related behaviors of subjects according to meal-skipping patterns

The analysis revealed significant differences in gender, age, marital status, education level, income, residential area, smoking, and drinking among meal-skipping patterns (Table 1; *p* < 0.05). The NS and SB groups had a higher proportion of men (52.18%, 56.60%), while the SD group had a higher proportion of women (69.83%). The NS group had a higher average age (53.43 ± 0.18), a higher percentage of high-income earners (27.73%), and rural residents (17.84%). The SB group had the lowest average age (38.26 ± 0.18) and the highest percentage of unmarried individuals (36.75%) and urban residents (88.67%). This group also had the highest percentage of individuals who engaged in heavy smoking (46.59%) and heavy drinking (28.77%), indicating an association with unhealthy lifestyles. The SD group had the lowest percentage of high-income earners (22.89%), heavy smokers (31.43%), and heavy drinkers (22.27%). There was no significant difference in exercise among the groups (*p* = 0.08).

**Table 1:** General characteristics according to meal-skipping patterns of the adults in South Korea (KNHANES 2013–2020)

Variables	No Skipping (*N* = 21,314)	Skipping Breakfast (*N* = 6,836)	Skipping Dinner (*N* = 380)	*P* value^8)^
Age (years) ^1)^	53.43 ± 0.18 ^a^	38.26 ± 0.18 ^c^	49.92 ± 0.92 ^b^	**< 0.0001**
Young (19–44y)	28.48 (0.50)	69.60 (0.68)	42.49 (3.09)	
Middle-aged (45–64y)	44.84 (0.46)	27.52 (0.66)	42.28 (2.97)	
Elderly (≥ 65y)	26.68 (0.48)	2.88 (0.20)	15.24 (1.87)	
Gender (%) ^2)^				**< 0.0001**
Male	52.18 (0.37)	56.60 (0.63)	30.17 (2.89)	
Female	47.82 (0.37)	43.40 (0.63)	69.83 (2.89)	
Marital status (%)				**< 0.0001**
Married	87.30 (0.34)	63.22 (0.82)	77.18 (2.80)	
Unmarried	12.67 (0.34)	36.75 (0.82)	22.82 (2.80)	
Graduation (%)				**< 0.0001**
Elementary	20.05 (0.45)	3.97 (0.25)	13.96 (1.76)	
Middle	10.74 (0.27)	4.96 (0.31)	10.46 (1.75)	
High	32.87 (0.46)	40.26 (0.76)	40.20 (3.07)	
University	36.33 (0.64)	50.82 (0.82)	35.38 (2.96)	
Household income (%) ^3)^				**< 0.0001**
Low	22.57 (0.48)	24.90 (0.76)	24.89 (2.64)	
Middle-Low	24.38 (0.43)	25.69 (0.70)	25.30 (2.78)	
Middle-High	25.32 (0.42)	26.14 (0.71)	26.91 (2.62)	
High	27.73 (0.59)	23.27 (0.75)	22.89 (2.63)	
Residential area (%) ^4)^				**< 0.0001**
Urban	82.16 (0.99)	88.67 (0.89)	83.38 (2.45)	
Rural	17.84 (0.99)	11.33 (0.89)	16.62 (2.45)	
Smoking (%) ^5)^				**< 0.0001**
Never	58.16 (0.39)	50.26 (0.69)	65.03 (2.95)	
Moderate	2.26 (0.14)	3.15 (0.26)	3.54 (1.19)	
High	39.58 (0.39)	46.59 (0.69)	31.43 (2.86)	
Drinking alcohol (%) ^6)^				**< 0.0001**
Never	19.52 (0.37)	10.88 (0.43)	15.42 (2.20)	
Moderate	56.96 (0.46)	60.36 (0.73)	62.31 (3.12)	
High	23.52 (0.40)	28.77 (0.68)	22.27 (2.68)	
Regular exercising (%) ^7)^				0.0812
Yes	47.08 (0.51)	48.75 (0.78)	51.16 (3.20)	
No	52.92 (0.51)	51.25 (0.78)	48.84 (3.20)	

^1)^Continuous variables are presented as mean ± standard error. Statistical analysis used proc surveyreq for significant differences between variables. Letters in different superscripts in the same row indicate values that are different at *p* < 0.05 according to the Bon-ferroni post-hoc tests

^2)^Categorical variables are presented as percentage distribution (standard error of percentage). Statistical analysis used Chi-square test for significant differences between variables

^3)^Income was divided into quartiles of individual income

^4)^Residential area was classified into two groups: Urbal area (Dong) and rural area (Eup-Myeon).

^5)^Smoking was categorized into Never, Moderate (<5 packs/d), and High (≥ 5 packs/d)

^6)^Drinking was categorized into None (None in the last year), Moderate (1 ~ 4 times a month), and High (≥ twice a week)

^7)^At least 2 h and 30 min of medium-intensity physical activity per week or at least 1 h and 15 min of high-intensity physical activity

^8)^Values in boldface are significant at *p*-value < 0.05

### Anthropometrics and biochemistry according to meal-skipping patterns

The analysis examined anthropometric measurements and blood test results for each meal-skipping pattern (Table 2). Among “young” adults, those who skipped breakfast (SB) had higher BMI, waist circumference, blood pressure, FBG, ALT, LDL-C, TC, and TG compared to non-skippers (NS). Conversely, in this age group, individuals who skipped dinner (SD) had lower blood pressure and higher HDL-C than SB. In “middle-aged” adults, SB was associated with higher WC, BMI, DBP, ALT, LDL-C, TC, and TG compared to NS, while SD was linked to lower WC, ALT, TG, and higher HDL-C. Among the “elderly” group, SB was associated with higher DBP, LDL-C, TC, and TG compared to NS, and SD was associated with higher BMI than SB. Across all age groups, there were significant differences in hyperglycemia prevalence based on meal-skipping patterns, with lipid parameters (except HDL-C) highest in the SB group. Additionally, the association between meal-skipping patterns and body measurements or biochemical indicators weakened as age increased.

**Table 2:** Anthropometrics and Biochemistry according to meal-skipping patterns of adults in South Korea (KNHANES 2013–2020) ^1)^

Variables	Young (19–44y) (*N* = 9,013)										Middle-aged (45–64y) (*N* = 11,168)										Elderly (≥ 65y) (*N* = 8,349)									
	NS (*N* = 4,515)			SB (*N* = 4,370)			SD (*N* = 128)			*P* value^2)^	NS (*N* = 8,880)			SB (*N* = 2,126)			SD (*N* = 162)			*P* value	NS (*N* = 7,919)			SB (*N* = 340)			SD (*N* = 90)			*P* value
Anthropometrics																														
Height (cm)	167.88	±	0.14 ^b^	169.25	±	0.14 ^a^	166.20	±	0.82 ^b^	**< 0.0001**	163.80	±	0.10 ^b^	164.42	±	0.20 ^a^	160.77	±	0.71 ^c^	**< 0.0001**	158.93	±	0.12	157.85	±	0.55	157.04	±	0.88	0.0186
Weight (kg)	66.55	±	0.24 ^b^	68.44	±	0.25 ^a^	66.67	±	1.52 ^ab^	**< 0.0001**	64.89	±	0.13 ^b^	66.07	±	0.28 ^a^	61.59	±	0.82 ^c^	**< 0.0001**	60.62	±	0.14	61.12	±	0.62	62.00	±	1.41	0.4589
WC (cm)	80.04	±	0.19 ^b^	81.13	±	0.20 ^a^	80.37	±	1.11 ^ab^	**0.0004**	83.39	±	0.12 ^b^	83.87	±	0.23 ^a^	81.34	±	0.73 ^a^	**0.0023**	85.76	±	0.13	87.01	±	0.58	86.53	±	1.55	0.0986
BMI (kg/m^2^)	23.45	±	0.07 ^b^	23.74	±	0.07 ^a^	23.98	±	0.40 ^ab^	**0.0121**	24.10	±	0.04 ^b^	24.33	±	0.08 ^a^	23.77	±	0.21 ^ab^	**0.0047**	23.96	±	0.04 ^b^	24.48	±	0.19 ^b^	25.14	±	0.48 ^a^	**0.0019**
SBP (mmHg)	111.08	±	0.22 ^b^	112.40	±	0.22 ^a^	109.12	±	1.24 ^b^	**< 0.0001**	119.47	±	0.21	119.53	±	0.39	118.91	±	1.43	0.9137	128.50	±	0.26	127.56	±	0.97	130.04	±	2.28	0.4967
DBP (mmHg)	74.24	±	0.18 ^b^	75.52	±	0.19 ^a^	73.53	±	0.90 ^b^	**< 0.0001**	78.09	±	0.13 ^b^	79.49	±	0.25 ^a^	77.74	±	1.04 ^ab^	**< 0.0001**	72.20	±	0.15 ^b^	74.23	±	0.58 ^a^	72.66	±	1.47 ^ab^	**0.0023**
**Biochemistry**																														
FBG (mg/dL)	93.70	±	0.29 ^b^	94.85	±	0.32 ^a^	91.83	±	2.06 ^ab^	**0.0123**	103.70	±	0.32	104.25	±	0.66	99.32	±	2.39	0.1280	107.95	±	0.36	108.95	±	2.17	105.21	±	3.15	0.6207
HbA1c (%)	5.47	±	0.01	5.40	±	0.01	5.44	±	0.07	0.1028	5.86	±	0.01	5.81	±	0.02	5.75	±	0.08	0.0679	6.08	±	0.01	6.08	±	0.06	6.01	±	0.10	0.7860
AST (IU/L)	21.43	±	0.20	22.17	±	0.28	21.23	±	0.93	0.0843	24.45	±	0.17	24.28	±	0.46	22.99	±	0.82	0.2156	24.73	±	0.14	23.46	±	0.56	26.09	±	1.60	0.0653
ALT (IU/L)	22.53	±	0.35 ^b^	24.66	±	0.45 ^a^	19.95	±	1.71 ^ab^	**< 0.0001**	23.03	±	0.21 ^a^	24.01	±	0.45 ^a^	20.23	±	1.06 ^b^	**0.0019**	20.63	±	0.16	19.42	±	0.59	20.36	±	1.21	0.1448
LDL-C (mg/dL)	113.85	±	1.10 ^b^	117.82	±	1.07 ^a^	109.63	±	6.20 ^ab^	**0.0173**	115.65	±	0.81 ^b^	119.92	±	1.48 ^a^	120.98	±	6.92 ^ab^	**0.0291**	107.35	±	0.93 ^b^	117.98	±	4.13 ^a^	112.54	±	8.07 ^ab^	**0.033**
HDL-C (mg/dL)	52.64	±	0.21 ^b^	52.06	±	0.20 ^b^	54.36	±	1.30 ^a^	**0.0308**	50.44	±	0.16 ^b^	50.82	±	0.31 ^b^	55.35	±	1.46 ^a^	**0.0024**	48.24	±	0.17	48.70	±	0.77	48.58	±	1.32	0.8261
TC (mg/dL)	184.85	±	0.58 ^b^	189.39	±	0.63 ^a^	189.22	±	3.63 ^ab^	**< 0.0001**	195.55	±	0.48 ^b^	202.06	±	0.91 ^a^	205.69	±	4.00 ^ab^	**< 0.0001**	181.97	±	0.54 ^b^	197.10	±	2.67 ^a^	188.49	±	6.07 ^ab^	**< 0.0001**
TG (mg/dL)	121.05	±	1.92 ^b^	134.45	±	2.11 ^a^	131.51	±	16.63 ^ab^	**< 0.0001**	148.52	±	1.67 ^b^	159.19	±	3.57 ^a^	131.17	±	9.47 ^b^	**0.0027**	130.98	±	1.16 ^b^	151.92	±	7.11 ^a^	133.29	±	9.06 ^ab^	**0.015**
**Hyperglycemia (%, SE)** ^3)^																														
Prediabetes ^4)^	14.22 (0.52)			18.28 (0.58)			12.50 (2.92)			**< 0.0001**	28.91 (0.48)			33.63 (1.02)			22.84 (3.30)			**< 0.0001**	29.33 (0.51)			32.94 (2.55)			23.33 (4.46)			**< 0.0001**
Diabetes ^5)^	3.10 (0.26)			2.93 (0.26)			1.56 (1.10)			**< 0.0001**	14.39 (0.37)			11.81 (0.70)			9.88 (2.34)			**< 0.0001**	26.51 (0.50)			22.94 (2.28)			17.78 (4.03)			**< 0.0001**

Abbreviations: KNHANES, Korea National Health and Nutrition Examination Survey; NS, no skipping; SB, skipping breakfast; SD, skipping dinner; WC, waist circumference; BMI, body mass index; SBP, systolic blood pressure; DBP, diastolic blood pressure; FBG, fasting blood glucose; HbA1c, glycated hemoglobin; TC, total cholesterol; TG, triglycerides; LDL-C, low density lipoprotein-cholesterol; HDL-C, high density lipoprotein-cholesterol; AST, aspartate transaminase; ALT, alanine transaminase

^1)^Variables are presented as mean ± standard error. Statistical analysis used proc surveyreq for significant differences between continuous variables

^2)^Values in boldface are significant at p-value < 0.05. Letters in different superscripts in the same row indicate values that are different at *p* < 0.05 according to the Bon-ferroni post-hoc tests

^3)^Categorical variables are presented as percentage distribution (standard error of percentage). Statistical analysis used Chi-square test for significant differences between variables

^4)^100 ≤ fasting blood glucose ≤ 125 or 5.7 ≤ glycated hemoglobin ≤ 6.4

^5)^126 ≤ fasting blood glucose or 6.5 ≤ glycated hemoglobin or taking diabetes medication or insulin injection or doctor’s diagnosis

### Comparison of daily nutrients intake levels according to meal-skipping patterns

Daily nutrient intake levels were assessed in relation to meal-skipping patterns within each age group (Table 3). Across all age categories, individuals in the non-skipping (NS) group exhibited the highest total daily calorie intake and calories per kilogram of body weight. However, in both the “young” and “middle-aged” groups, those who skipped breakfast (SB) had the highest ratios of energy intake from fat (% of energy; young: 25.3%; middle-aged: 20.7%) and saturated fatty acids (SFA) (young: 8.41%; middle-aged: 6.62%), as well as the highest ratio of omega-6(n-6) to omega-3(n-3) fatty acids among all age-groups (n-6/n-3; young: 8.41; middle-aged: 7.52; elderly: 7.24). Conversely, when examining micronutrient intake per 1,000 calories to determine the density of daily micronutrient consumption, individuals who skipped dinner (SD) consistently demonstrated the highest intake levels of calcium, phosphorus, and potassium per 1,000 calories across all age groups. To summarize, the analysis highlighted elevated consumption of SFA and n-6 fatty acids in the SB group, while emphasizing the superior nutrient density of calcium, phosphorus, and potassium in the SD group across different age categories.

**Table 3:** Daily intakes of the subjects according to meal-skipping patterns of adults in South Korea (KNHANES 2013–2020) ^1)^

Variables	Young (19–44y) (*N* = 9,013)									*P* value^2)^	Middle-aged (45–64y) (*N* = 11,168)									*P* value	Elderly (≥ 65y) (*N* = 8,349)									*P* value
	NS (*N* = 4,515)			SB (*N* = 4,370)			SD (*N* = 128)				NS (*N* = 8,880)			SB (*N* = 2,126)			SD (*N* = 162)				NS (*N* = 7,920)			SB (*N* = 340)			SD (*N* = 90)			
Energy (kcal)	2213.34	±	14.97^a^	2141.76	±	15.09^b^	1741.18	±	76.38^c^	**< 0.0001**	2093.78	±	10.90^a^	1885.46	±	19.23^b^	1719.97	±	80.29^b^	**< 0.0001**	1722.42	±	10.37^a^	1419.09	±	37.40^b^	1507.99	±	75.73 ^b^	**< 0.0001**
Energy (kcal/kg)	33.91	±	0.22^a^	32.10	±	0.23^b^	27.10	±	1.29^c^	**< 0.0001**	32.61	±	0.16^a^	28.94	±	0.29^b^	28.13	±	1.21^b^	**< 0.0001**	28.73	±	0.17^a^	23.64	±	0.64^b^	24.57	±	1.19 ^b^	**< 0.0001**
**Macronutrients**																														
**C : P : F (% of energy)**	61.3 : 15.6 : 23.1			58.7 : 16.0 : 25.3			61.0 : 15.3 : 23.7				66.1 : 14.9 : 19.0			64.1 : 15.3 : 20.7			64.8 : 15.0 : 20.2				71.7 : 13.7 : 14.7			71.4 : 13.3 : 15.3			69.2 : 13.9 : 16.9			
Carbohydrate (g)	319.01	±	2.15^a^	287.51	±	2.02^b^	250.16	±	11.38^c^	**< 0.0001**	323.43	±	1.65^a^	274.92	±	2.83^b^	255.12	±	9.42^b^	**< 0.0001**	295.96	±	1.72^a^	239.33	±	6.35^b^	248.99	±	11.38^b^	**< 0.0001**
Protein (g)	82.61	±	0.70^a^	79.47	±	0.70^b^	61.92	±	3.15^c^	**< 0.0001**	74.35	±	0.46^a^	66.59	±	0.85^b^	58.51	±	2.49^c^	**< 0.0001**	57.74	±	0.45^a^	45.63	±	1.53^b^	52.71	±	4.47^a, b^	**< 0.0001**
Protein (g/kg)	1.26	±	0.01^a^	1.19	±	0.01^b^	0.96	±	0.05^c^	**< 0.0001**	1.16	±	0.01^a^	1.02	±	0.01^b^	0.96	±	0.04^b^	**< 0.0001**	0.96	±	0.01^a^	0.76	±	0.03^b^	0.85	±	0.07^a, b^	**< 0.0001**
Fat (g)	56.09	±	0.61^a^	58.00	±	0.65^a^	44.62	±	2.98^b^	**< 0.0001**	43.32	±	0.38^a^	41.70	±	0.74^a, b^	36.87	±	2.16^b^	**< 0.0001**	28.42	±	0.37^b^	24.86	±	1.30^a^	28.03	±	2.19^a, b^	**0.0286**
**SFA : MUFA : PUFA**	1.28 : 1.33 : 1.00			1.34 : 1.38 : 1.00			1.40 : 1.33 : 1.00				1.10 : 1.17 : 1.00			1.22 : 1.25 : 1.00			1.15 : 1.15 : 1.00				1.01 : 1.01 : 1.00			1.12 : 1.12 : 1.00			1.14 : 1.06 : 1.00			
SFA (% of energy)	7.46	±	0.06^b^	8.41	±	0.07^a^	8.04	±	0.46^a, b^	**< 0.0001**	5.82	±	0.04^b^	6.62	±	0.10^a^	6.43	±	0.31^a, b^	**< 0.0001**	4.43	±	0.04^b^	4.87	±	0.22^a, b^	5.64	±	0.47^a^	**0.0043**
**N-6 : N-3**	7.76 : 1.00 ^b^			8.41 : 1.00 ^a^			8.03 : 1.00^a, b^			**< 0.0001**	6.48	±	1.00^b^	7.25 : 1.00^a^			6.58 : 1.00^a, b^			**< 0.0001**	6.12 : 1.00^b^			7.24 : 1.00^a^			6.83 : 1.00 ^a, b^			**0.0003**
**Micronutrients (/1,000kcal)** ^3)^																														
Calcium (mg)	255.23	±	2.23^c^	241.06	±	2.25^b^	303.37	±	15.78^a^	**< 0.0001**	280.01	±	1.85^b^	262.47	±	3.33^c^	328.90	±	19.65^a^	**< 0.0001**	272.06	±	2.27^b^	257.16	±	8.10^b^	345.83	±	28.91^a^	**0.0082**
Phosphorus (mg)	549.82	±	2.44^a^	523.91	±	2.35^b^	570.33	±	16.69^a^	**< 0.0001**	565.79	±	1.90^a^	544.40	±	3.68^b^	593.10	±	16.28^a^	**< 0.0001**	547.36	±	2.20^a^	524.79	±	7.75^b^	597.77	±	21.99^a^	**0.0012**
Iron (mg)	7.00	±	0.06^a^	6.41	±	0.08^b^	6.56	±	0.28^a, b^	**< 0.0001**	7.66	±	0.10^a^	6.73	±	0.09^b^	7.83	±	0.52^a, b^	**< 0.0001**	7.53	±	0.07	7.08	±	0.20	6.97	±	0.44	0.0379
Sodium (mg)	1825.60	±	14.31	1858.38	±	14.18	1795.35	±	81.81	0.2028	1863.26	±	11.16	1859.58	±	22.28	1737.95	±	71.43	0.2211	1790.74	±	13.64	1794.21	±	61.80	1634.69	±	107.44	0.3567
Potassium (mg)	1443.47	±	8.23^a^	1309.55	±	7.28^b^	1532.43	±	78.24^a^	**< 0.0001**	1630.18	±	7.82^a^	1543.84	±	13.26^b^	1736.43	±	68.90^a^	**< 0.0001**	1621.43	±	9.39^b^	1554.79	±	32.98^a, b^	1832.19	±	93.92^a^	**0.0108**
Vitamin A (µgREl)	332.34	±	5.57^a^	293.10	±	5.01^b^	336.71	±	22.54^a, b^	**< 0.0001**	370.71	±	6.21^a^	336.91	±	12.27^b^	380.64	±	31.21^a, b^	**0.0416**	342.21	±	7.46	355.17	±	4.01	439.62	±	69.24	0.3649
Carotene (µg)	1508.51	±	27.36^a^	1279.48	±	21.05^b^	1365.38	±	116.98^a, b^	**< 0.0001**	1828.64	±	27.40^a^	1646.72	±	49.55^b^	1770.11	±	135.02^a, b^	**0.0050**	1796.39	±	35.76	1733.02	±	205.70	2139.76	±	288.29	0.4697
Retinol (µg)	75.97	±	2.08	78.36	±	2.76	92.00	±	10.47	0.2654	62.73	±	1.93	60.03	±	2.47	79.49	±	8.60	0.0884	46.58	±	1.52	41.68	±	4.01	86.95	±	19.03	0.0536
Vitamin B1 (mg)	0.82	±	0.01^a^	0.77	±	0.01^b^	0.77	±	0.03^a, b^	**< 0.0001**	0.82	±	0.00^a^	0.76	±	0.01^b^	0.80	±	0.04^a, b^	**< 0.0001**	0.79	±	0.00^a^	0.74	±	0.02^b^	0.75	±	0.04^a, b^	**0.0030**
Vitamin B2 (mg)	0.80	±	0.01	0.81	±	0.01	0.87	±	0.04	0.0484	0.78	±	0.00	0.79	±	0.01	0.89	±	0.05	0.0397	0.70	±	0.01	0.70	±	0.02	0.80	±	0.04	0.0605
Niacin (mg)	7.66	±	0.06^a^	7.38	±	0.06^b^	7.80	±	0.33^a, b^	**0.0025**	7.31	±	0.03	7.17	±	0.07	7.23	±	0.26	0.1950	6.65	±	0.04	6.42	±	0.14	6.93	±	0.37	0.1953
Vitamin C (mg)	39.05	±	0.76^a^	32.01	±	0.78^b^	47.36	±	6.60^a, b^	**< 0.0001**	45.86	±	0.72^a^	39.25	±	1.29^b^	57.38	±	7.99^a, b^	**< 0.0001**	42.06	±	0.69	44.22	±	4.20	62.17	±	10.24	0.1221

Abbreviations: KNHANES, Korea National Health and Nutrition Examination Survey; NS, no skipping; SB, skipping breakfast; SD, skipping dinner; SFA, saturated fatty acids; MUFA, mono unsaturated fatty acids; PUFA, poly unsaturated fatty acids; N-6, omega-6 fatty acid; N-3, omega-3 fatty acid

^1)^Variables are presented as mean ± standard error. Statistical analysis used proc surveyreq for significant differences between continuous variables

^2)^Values in boldface are significant at *p*-value < 0.05. Letters in different superscripts in the same row indicate values that are different at *p* < 0.05 according to the Bon-ferroni post-hoc tests

^3)^Micronutrients are expressed as intakes per 1,000 kcal

### Comparison of the quality of meals according to meal-skipping patterns

**Fig. 1:**
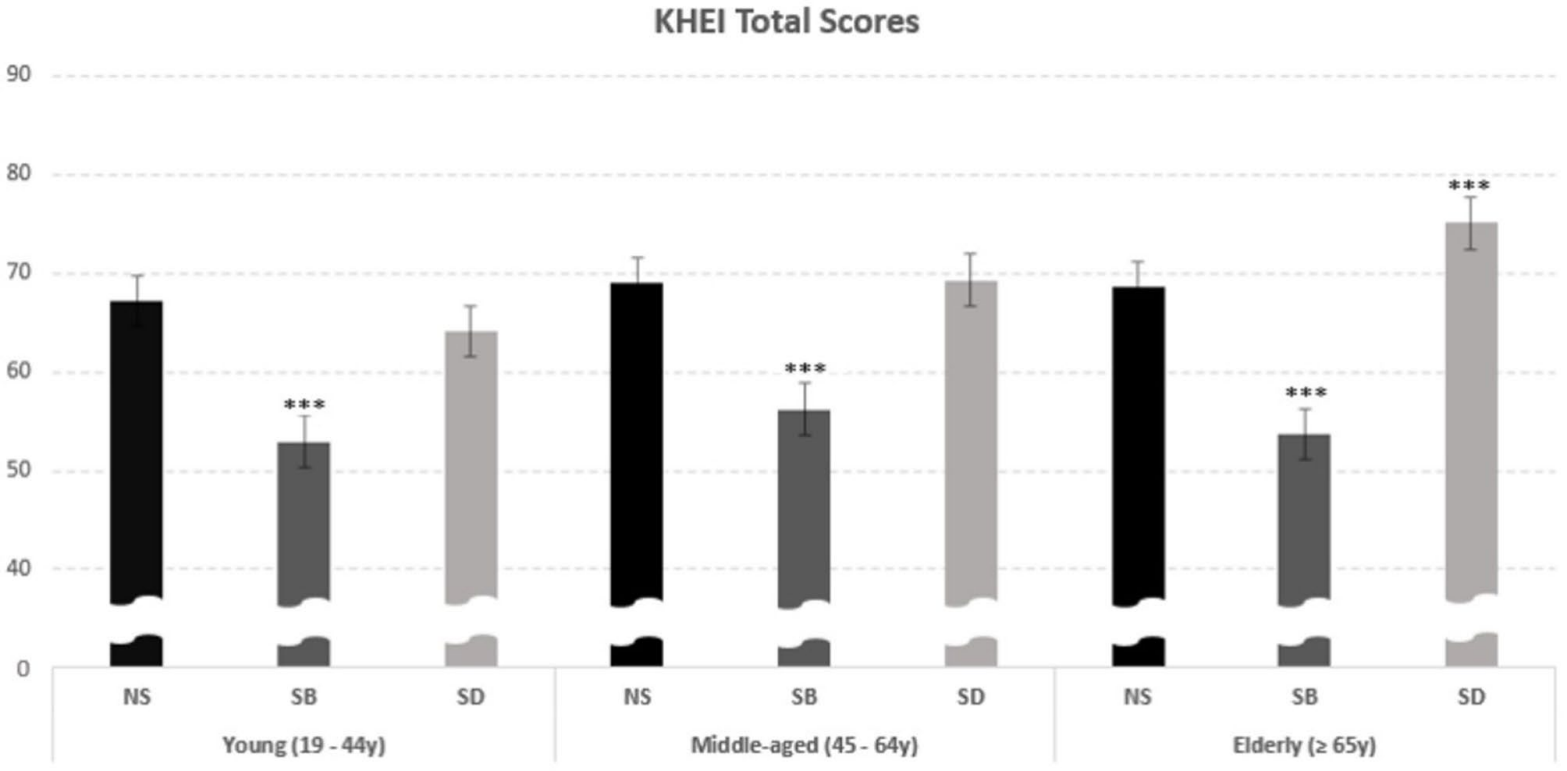
Comparison of KHEI scores representing meal quality between meal-skipping patterns within the same age group. Statistical analysis was performed using proc surveyreq to find the significant difference between variables, and *** indicates *p* < 0.001 when compared with NS of the same age group using the Bon-ferroni post hoc test. Abbreviations: KHEI, Korean Health Eating Index; NS, no skipping; SB, skipping breakfast; SD, skipping dinner

The assessment of meal quality utilized KHEI scores, eating habits surveys, and selected food group intakes as indicators. Notably, the total KHEI score, which evaluates dietary quality, was significantly lower in the group that skipped breakfast (SB) across all age groups (Young, Middle-aged, Elderly: 52.95, 56.19, 53.66, *p* < 0.05) (Fig. 1). Remarkably, in the “elderly” group, the total KHEI score for those who skipped dinner (SD) was 75.06 points, significantly higher than the score of 68.59 for non-skippers (NS). Consequently, this assessment highlights that meal quality was the poorest among those who skipped breakfast (SB). Interestingly, for the “elderly” group, meal quality in the SD group was superior to that in the NS group. For additional details, please refer to supplemental Table 1.

Additionally, across all age groups, the frequency of eating out more than three times a week was notably high (Young, Middle-aged, Elderly: 78.35%, 61.25%, and 24.17%) among those who skipped breakfast (SB), although this difference was not significant in the “elderly” group (Table 4). Furthermore, the dietary intake of meat, beverages, and alcohol was significantly higher in the SB group compared to non-skippers (NS), but this significance was observed primarily in the “young” and “middle-aged” groups. In the “elderly” group, while the intake of beverages and alcohol was highest in the SB group, this difference was not significant (*p* > 0.05). Additionally, milk intake was highest in the group that skipped dinner (SD) across all age groups, except for the young group where this difference was not significant.

**Table 4:** Eating habits survey and Selected food groups intakes of the subjects according to meal-skipping patterns of adults in South Korea (KNHANES 2013–2020)

Variables	Young (19–44y) (*N* = 9,013)									*P* value^4)^	Middle-aged (45–64y) (*N* = 11,168)									*P* value	Elderly (≥ 65y) (*N* = 8,349)									*P* value
	NS (*N* = 4,515)			SB (*N* = 4,370)			SD (*N* = 128)				NS (*N* = 8,880)			SB (*N* = 2126)			SD (*N* = 162)				NS (*N* = 7,919)			SB (*N* = 340)			SD (*N* = 90)			
**Eating habits survey** ^1)^																														
Eat out more than three times a week	71.26 (0.77)			78.35 (0.75)			51.44 (5.13)			**< 0.0001**	54.10 (0.66)			61.25 (1.26)			35.28 (4.40)			**< 0.0001**	19.04 (0.62)			24.17 (2.72)			20.65 (4.67)			0.1030
Have experience in nutrition education	4.83 (0.35)			3.71 (0.30)			2.42 (1.28)			**0.0238**	4.66 (0.28)			3.25 (0.44)			8.56 (2.42)			**0.0036**	6.19 (0.35)			4.73 (1.17)			3.80 (2.42)			0.4309
Use nutritional labeling	44.30 (0.88)			35.60 (0.85)			51.49 (5.16)			**< 0.0001**	29.65 (0.66)			30.26 (1.32)			38.08 (4.96)			0.2206	19.17 (0.84)			25.16 (4.16)			13.15 (5.43)			0.1777
Whether others have accompanied you during breakfast in the last year	61.25 (0.88)			-^3)^			54.43 (5.00)			0.1696	60.89 (0.65)			-			54.62 (4.58)			0.1674	64.94 (0.72)			-			52.48 (6.54)			**0.0475**
Whether others have accompanied you during lunch in the last year	75.09 (0.71)			74.69 (0.75)			54.45 (5.13)			**< 0.0001**	69.89 (0.57)			70.58 (1.11)			61.36 (4.51)			0.1111	60.43 (0.71)			43.78 (3.17)			43.01 (5.98)			**< 0.0001**
Whether others have accompanied you during dinner in the last year	85.78 (0.64)			82.37 (0.71)			-			**0.0002**	80.15 (0.54)			80.15 (0.54)			-			**0.0025**	70.99 (0.69)			53.69 (3.05)			-			**< 0.0001**
**Selected food groups** ^2)^																														
Grains (g)	318.68	±	2.90^a^	290.06	±	2.81^b^	254.48	±	16.66^c^	**< 0.0001**	303.11	±	2.06^a^	257.85	±	3.70^b^	229.61	±	12.90^c^	**< 0.0001**	281.79	±	1.96^a^	231.14	±	8.15^b^	212.80	±	17.76^b^	**< 0.0001**
Sugars (g)	11.83	±	0.35	11.58	±	0.35	11.64	±	2.79	0.8799	9.79	±	0.24	8.85	±	0.39	7.60	±	1.28	0.0481	7.12	±	0.20	7.51	±	1.03	6.71	±	1.57	0.8987
Legumes (g)	37.17	±	1.38^a^	29.25	±	1.41^b^	31.00	±	6.73^a, b^	**0.0003**	44.24	±	1.05^a^	32.08	±	1.59^b^	43.92	±	8.10^a, b^	**< 0.0001**	45.12	±	1.19^a^	34.95	±	4.34^a, b^	30.04	±	5.31^b^	**0.0027**
Vegetables (g)	311.69	±	3.57^a^	262.09	±	3.16^b^	232.16	±	21.83^b^	**< 0.0001**	379.05	±	3.19^a^	301.44	±	4.92^b^	297.95	±	17.88^b^	**< 0.0001**	333.90	±	3.48^a^	241.35	±	11.59^b^	267.24	±	23.59^b^	**< 0.0001**
Fruits (g)	164.49	±	4.00^a^	103.88	±	3.99^b^	148.31	±	24.23^a, b^	**< 0.0001**	226.74	±	3.97^a^	175.70	±	6.85^b^	233.60	±	28.26^a, b^	**< 0.0001**	186.99	±	3.88	162.25	±	17.74	248.80	±	34.71	0.0750
Meats (g)	147.28	±	3.04^b^	162.09	±	2.91^a^	84.08	±	12.40^c^	**< 0.0001**	96.18	±	1.72^a^	106.91	±	4.26^a^	68.76	±	10.46^b^	**0.0016**	62.83	±	1.80^a^	60.35	±	7.43	41.10	±	8.00^b^	0.0303
Eggs (g)	36.48	±	0.93^a^	31.52	±	0.80^b^	36.38	±	5.19^a, b^	**0.0002**	31.12	±	0.65	28.27	±	1.15	28.21	±	3.81	**0.0801**	20.01	±	0.51	16.36	±	2.19	19.32	±	4.37	0.2638
Fishes (g)	102.56	±	2.68^a^	91.60	±	2.35^b^	63.05	±	8.68^c^	**< 0.0001**	126.91	±	2.50^a^	102.28	±	4.06^b^	79.66	±	11.02^b^	**< 0.0001**	102.14	±	2.56 ^a^	76.76	±	8.12	97.32	±	18.64^a, b^	**0.0116**
Milk (g)	106.36	±	2.95^a^	90.79	±	2.79^b^	140.14	±	20.76^a, b^	**< 0.0001**	79.31	±	1.86^a^	62.49	±	3.07^b^	115.98	±	15.97^b^	**< 0.0001**	60.49	±	1.80 ^a^	43.76	±	43.76	132.70	±	19.91^c^	**< 0.0001**
Beverages (g)	233.00	±	5.49^b^	318.21	±	6.73^a^	252.54	±	35.08^a, b^	**< 0.0001**	150.73	±	3.28^b^	186.27	±	7.11^a^	192.11	±	48.85^a, b^	**< 0.0001**	58.74	±	1.99	71.50	±	9.15	73.89	±	20.67	0.3086
Alcohol (g)	121.56	±	6.20^b^	184.52	±	7.88^a^	95.81	±	24.02^b^	**< 0.0001**	123.90	±	4.49^b^	158.24	±	10.21^a^	138.39	±	41.76^a, b^	**0.0084**	49.61	±	2.60	61.63	±	14.72	45.09	±	23.94	0.7123

Abbreviations: KNHANES, Korea National Health and Nutrition Examination Survey; NS, no skipping; SB, skipping breakfast; SD

^1)^Categorical variables are presented as percentage distribution (standard error of percentage). Statistical analysis used Chi-square test for significant differences between variables

^2)^Continuous variables are presented as mean ± standard error. Statistical analysis used proc surveyreq for significant differences between variables

^3)^No observations for that value

^4)^Values in boldface are significant at *p*-value < 0.05. Letters in different superscripts in the same row indicate values that are different at *p* < 0.05 according to the Bon-ferroni post-hoc tests

### Association between meal-skipping patterns and hyperglycemia

**Fig. 2:**
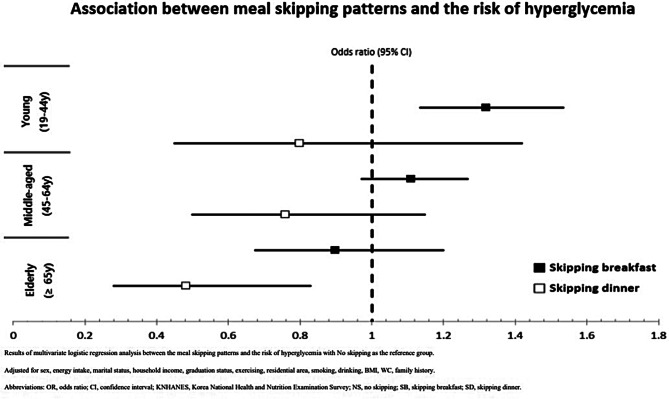
Association between meal-skipping patterns and the risk of hyperglycemia in Korean adults by age group (KNHANES 2013–2020). Results of multivariable logistic regression analysis between the meal-skipping patterns and the risk of hyperglycemia with no skipping as the reference group. Adjusted for sex, energy intake, marital status, household income, graduation status, exercising, residential area, smoking, drinking, BMI, family history. Abbreviations: OR, odds ratio; CI, confidence interval; KNHANES, Korea National Health and Nutrition Examination Survey; NS, no skipping; SB, skipping breakfast; SD, skipping dinner

The results of the multivariable logistic regression analysis unveiled significant associations between meal-skipping patterns and the risk of hyperglycemia, as illustrated in Fig. 2. In the “young” group, skipping breakfast (SB) was found to significantly increase the risk of hyperglycemia. Even after adjusting for various factors including sex, energy intake, marital status, income, education level, exercise, residential area, smoking, drinking, BMI, and family history, SB was associated with a 1.327 times higher risk of hyperglycemia (95% CI 1.142–1.544). However, there was no significant association between skipping dinner (SD) and hyperglycemia in this group. In the “middle-aged” group, neither SB nor SD exhibited significant associations with hyperglycemia when all variables were adjusted. Conversely, in the “elderly” group, there was no significant association between SB and hyperglycemia in any of the adjusted models. However, SD displayed a significant inverse association with hyperglycemia across all adjusted models. After accounting for all covariates, the risk of hyperglycemia in SD was 0.487 times lower compared to non-skippers (NS) (95% CI 0.290–0.821). In summary, the results indicate a positive relationship between SB and the risk of hyperglycemia in the younger age group, while a inverse relationship between SD and hyperglycemia was observed in the age group of 65 years and older. For detailed results, please refer to supplementary Table 2.

## Discussion

This nationally representative cross-sectional study furnishes valuable insights into the intricate interplay between meal skipping patterns and the risk of hyperglycemia across various age groups. Our findings underscore that the act of skipping breakfast is closely linked to a 30% heightened susceptibility to hyperglycemia among adults aged 19–44. In contrast, among adults aged 65 or older who skipped dinner, a lower risk of hyperglycemia, approximately 50%, was observed.

Contrary to the well-known health benefits of intermittent fasting, a typical dietary pattern associated with meal skipping [23], this study confirmed that breakfast skipping was associated with elevated blood glucose. According to a study by Nas, et al. [24], skipping breakfast had higher blood glucose levels after lunch compared to skipping dinner. The authors explained that skipping breakfast can lead to impaired glucose homeostasis by developing metabolic inflexibility that increases fat oxidation despite increased insulin levels after meals. The exact effect of skipping meals on blood glucose is unknown, but several studies have shown that it may be due to disturbances in the body’s hormone regulatory system. Skipping breakfast has been linked to chronic stress in the hypothalamic-pituitary-adrenal (HPA) axis, leading to a sustained increase in cortisol levels [25]. This continuous elevation of cortisol is associated with insulin resistance [26]. Therefore, meal skipping could potentially interfere with the body’s hormonal regulatory system, contributing to the development of hyperglycemia.

However, the association between skipping breakfast and hyperglycemia was found only in the 19–44 age group. According to the population distribution by meal-skipping pattern, in contrast to the high distribution of NS in the “middle-aged” and “elderly” groups, about half of the population in the “young” group was skipping breakfast. In other age groups, results may not have been derived due to the relatively small number of samples within the group.

The disturbance of the regulatory system also affects the lipid profile. The prolonged elevation of cortisol is a factor associated with both hypertriglyceridemia and reduced levels of HDL-C [27]. Our study observed that all age groups exhibited elevated levels of TC, TG, and LDL-C when breakfast was skipped, compared to those who adhered to a regular meal schedule. This corroborates earlier research indicating that the practice of skipping breakfast is linked to an increased risk of dyslipidemia [28, 29]. The exact mechanism underlying the relationship between blood lipid levels and meal skipping remains uncertain. Nevertheless, the observed rise in triglyceride levels during meal skipping might be linked to increased calorie intake per each eating occasion [30].

Regarding body weight, despite the reduction in meal frequency, individuals who skipped meals did not demonstrate lower BMI compared to those who adhered to a regular meal schedule. It is consistent with the results of a meta-analysis that skipping breakfast can increase the risk of obesity [31]. This underscores the need for caution in advocating short-term meal reduction for weight loss. Indeed, the recent consensus statement by the Korean Society for the Study of Obesity, the Korean Diabetes Association, and the Korean Society of Hypertension refrained from endorsing time-restricted intermittent fasting in overweight or obese adults due to the scarcity of long-term studies and the heterogeneity of prior research findings [32].

In the “young” group, the SB with the highest BMI had a higher risk of hyperglycemia, whereas in the “elderly” group, the SD with the highest BMI had a lower risk of hyperglycemia. In “elderly” adults, the relationship between BMI and disease risk is less closely related than in younger people because BMI may be underestimated due to changes in body composition or overestimated due to decreased height due to spinal disease [33]. In addition to this, it may be due to the influence of other factors such as the composition of the meal. This underscores the imprudence of relying solely on biochemical parameters to determine diabetes risk in the elderly.

Another salient finding of our study pertains to the pivotal role of diet quality and composition in influencing the risk of hyperglycemia. Our results corroborate previous research [34] highlighting that the act of skipping breakfast can have a detrimental impact on overall dietary quality. Furthermore, this group displayed elevated consumption of meat, beverages, and alcohol, which previous studies have linked to an increased risk of type 2 diabetes [35–37]. Conversely, for the “elderly” group aged 65 and over, the SD showed the highest KHEI score, and the risk of hyperglycemia was low. Previous research has similarly highlighted that increased consumption of vegetables, fruits, milk, and dairy products is associated with a reduced prevalence of type 2 diabetes among Koreans [12, 38, 39]. This suggests that individuals with dinner skipping patterns tend to eat healthier than individuals with breakfast skipping patterns. This result should be careful not to be interpreted as meaning to skip dinner to manage blood glucose in the elderly population. Because the elderly population is at high risk of malnutrition due to difficulties in storage and loss of appetite, it is important to set up a strategy to ensure healthy eating rather than skipping meals to control blood glucose.

In this study, we identified several strengths. Firstly, it enhanced the statistical reliability of the research findings by utilizing the extensive KNHANES dataset, representing a substantial sample of the Korean population. Secondly, our study is the first comprehensive analysis of the correlation between skipping breakfast and dinner and hyperglycemia. Thirdly, we categorized Korean adults into young, middle-aged, and elderly groups, providing insights for the population aged 65 years or older, a demographic often overlooked in previous research. While prior studies targeting Westerners or Asians have shown that skipping breakfast adversely affects blood sugar control and increases the risk of type 2 diabetes [40, 41], none have been exclusively focused on the elderly. This study further underscores the importance of not just dietary content but also meal timing, potentially informing future dietary recommendations to mitigate hyperglycemia risk among Koreans.

Despite these advantages, this study has several limitations. This study was cross-sectional, limiting the ability to establish a clear causal relationship between dietary patterns and hyperglycemia. Additionally, although the survey was conducted by well-trained staff, information bias may occur because self-reported data was used. The limited sample size of the SD is acknowledged as a potential limitation in our study, which may affect the generalizability of the findings, necessitating careful interpretation of the results. Additionally, we were unable to fully separate the relationship between meal patterns and hyperglycemia from meal composition or quality.

In conclusion, this study suggests that the meal-skipping pattern, especially breakfast omission, affects the risk of hyperglycemia, which may show different results depending on the age group. This association was not explained except for the composition and quality of the meal. Further, longitudinal studies are needed to control the composition and quality of meals and focus on the meal-skipping pattern and hyperglycemia in order to determine the independent causal relationship of the meal-skipping pattern.

### Electronic supplementary material

Below is the link to the electronic supplementary material.

Supplementary Material 1

## Data Availability

The datasets generated and analyzed for the current study are available in the Korea National Health & Nutrition Examination Survey repository, https://knhanes.kdca.go.kr/.
