# Supplementary material for: Age-specific association between meal-skipping patterns and the risk of hyperglycemia in Korean adults: a national cross-sectional study using the KNHANES data
Source: BMC Public Health. 2024 Jun 25;24:1697. doi: 10.1186/s12889-024-18762-w (PMC11201090; doi:10.1186/s12889-024-18762-w)
Supplement: Supplementary file 1 — Supplementary Material 1 [file 12889_2024_18762_MOESM1_ESM.docx]

| **Supplemental table 1. Korean Health Eating Index of the subjects according to meal-skipping patterns of adults in South Korea (KNHANES 2013-2020)** | | | | | | | | | | | | | | | | | | | | | | | | | | | | | | |
| --- | --- | --- | --- | --- | --- | --- | --- | --- | --- | --- | --- | --- | --- | --- | --- | --- | --- | --- | --- | --- | --- | --- | --- | --- | --- | --- | --- | --- | --- | --- |
| **Variables** | **Young (19 - 44y)  (N=9,013)** | | | | | | | | | ***P* value** | **Middle-aged (45 - 64y)  (N=11,168)** | | | | | | | | | ***P* value** | **Young (≥ 65y)  (N=8,349)** | | | | | | | | | ***P* value** ^2^**^)^** |
|  | **NS (N=4,515)** | | | **SB (N=4,370)** | | | **SD (N=128)** | | |  | **NS (N=8,880)** | | | **SB (N=2,126)** | | | **SD (N=162)** | | |  | **NS (N=7,919)** | | | **SB (N=340)** | | | **SD (N=90)** | | |  |
| **Total Scores (0-100)** | 67.16 | ± | 0.22 ^a^ ^1)^ | 52.95 | ± | 0.24 ^b^ | 64.11 | ± | 1.34 ^a^ | **<.0001** | 69.02 | ± | 0.17 ^a^ | 56.19 | ± | 0.41 ^b^ | 69.32 | ± | 1.17 ^a^ | **<.0001** | 68.60 | ± | 0.22 ^b^ | 53.66 | ± | 0.93 ^c^ | 75.06 | ± | 1.71 ^a^ | **<.0001** |
| **Adequacy Component** |  |  |  |  |  |  |  |  |  |  |  |  |  |  |  |  |  |  |  |  |  |  |  |  |  |  |  |  |  |  |
| Have Breakfast (0-10) | 10.00 | ± | 0.00 ^a^ | 1.34 | ± | 0.04 ^b^ | 10.00 | ± | 0.00 ^a^ | **<.0001** | 10.00 | ± | 0.00 ^a^ | 1.30 | ± | 0.05 ^b^ | 10.00 | ± | 0.00 ^a^ | **<.0001** | 10.00 | ± | 0.00 ^a^ | 0.83 | ± | 0.10 ^b^ | 10.00 | ± | 0.00 ^a^ | **<.0001** |
| Whole grains (0-5) | 2.16 | ± | 0.04 ^a^ | 1.33 | ± | 0.04 ^b^ | 1.57 | ± | 0.23 ^b^ | **<.0001** | 2.54 | ± | 0.03 ^a^ | 1.88 | ± | 0.07 ^b^ | 2.73 | ± | 0.24 ^a^ | **<.0001** | 2.81 | ± | 0.04 ^a^ | 2.09 | ± | 0.17 ^b^ | 2.82 | ± | 0.30 ^a, b^ | **0.0001** |
| Fruits including juice (0-5) | 2.14 | ± | 0.04 ^a^ | 1.37 | ± | 0.04 ^b^ | 1.98 | ± | 0.24 ^a^ | **<.0001** | 2.64 | ± | 0.04 ^a^ | 2.09 | ± | 0.07 ^b^ | 2.87 | ± | 0.24 ^a^ | **<.0001** | 2.79 | ± | 0.04 ^b^ | 2.62 | ± | 0.19 ^b^ | 3.74 | ± | 0.34 ^a^ | **0.0140** |
| Fruits excluding juice (0-5) | 2.38 | ± | 0.05 ^a^ | 1.37 | ± | 0.04 ^b^ | 2.18 | ± | 0.27 ^a^ | **<.0001** | 2.87 | ± | 0.04 ^a^ | 2.3 | ± | 0.08 ^b^ | 3.13 | ± | 0.25 ^a^ | **<.0001** | 2.89 | ± | 0.04 | 2.72 | ± | 0.19 | 3.46 | ± | 0.41 | 0.2347 |
| Vegetables including Kimchi or pickles (0-5) | 3.57 | ± | 0.03 ^a^ | 3.21 | ± | 0.03 ^b^ | 2.70 | ± | 0.18 ^c^ | **<.0001** | 3.96 | ± | 0.02 ^a^ | 3.54 | ± | 0.05 ^b^ | 3.32 | ± | 0.16 ^b^ | **<.0001** | 3.80 | ± | 0.02 ^a^ | 3.06 | ± | 0.13 ^b^ | 3.89 | ± | 0.19 ^a^ | **<.0001** |
| Vegetables excluding Kimchi and pickles (0-5) | 3.32 | ± | 0.03 ^a^ | 2.95 | ± | 0.03 ^b^ | 2.59 | ± | 0.19 ^b^ | **<.0001** | 3.63 | ± | 0.02 ^a^ | 3.13 | ± | 0.05 ^b^ | 2.98 | ± | 0.18 ^b^ | **<.0001** | 3.48 | ± | 0.03 ^a^ | 2.68 | ± | 0.13 ^b^ | 3.72 | ± | 0.24 ^a^ | **<.0001** |
| Meat, fish, eggs and legumes (0-10) | 7.63 | ± | 0.05 ^a^ | 7.54 | ± | 0.06 ^a^ | 6.47 | ± | 0.32 ^b^ | **0.0012** | 7.10 | ± | 0.05 ^a^ | 6.65 | ± | 0.09 ^b^ | 6.41 | ± | 0.39 ^a, b^ | **<.0001** | 6.80 | ± | 0.03 ^a^ | 5.65 | ± | 0.27 ^b^ | 7.27 | ± | 0.49 ^a^ | **0.0001** |
| Milk and dairy (0-10) | 3.95 | ± | 0.09 ^a^ | 3.46 | ± | 0.10 ^b^ | 4.49 | ± | 0.59 ^a, b^ | **0.0005** | 3.15 | ± | 0.07 ^b^ | 2.61 | ± | 0.13 ^c^ | 4.55 | ± | 0.53 ^a^ | **<.0001** | 2.50 | ± | 0.07 ^b^ | 1.90 | ± | 0.29 ^b^ | 5.08 | ± | 0.68 ^a^ | **<.0001** |
| **Moderation Component** |  |  |  |  |  |  |  |  |  |  |  |  |  |  |  |  |  |  |  |  |  |  |  |  |  |  |  |  |  |  |
| Sodium (0-10) | 5.79 | ± | 0.07 ^a^ | 5.91 | ± | 0.07 ^a^ | 7.34 | ± | 0.33 ^b^ | **<.0001** | 6.04 | ± | 0.05 ^b^ | 6.69 | ± | 0.11 ^c^ | 7.57 | ± | 0.33 ^a^ | **<.0001** | 7.33 | ± | 0.05 ^a^ | 8.21 | ± | 0.23 ^b^ | 8.27 | ± | 0.33 ^b^ | **<.0001** |
| Saturated fatty acids (0-10) | 7.19 | ± | 0.08 ^a^ | 6.24 | ± | 0.09 ^b^ | 6.27 | ± | 0.52 ^a, b^ | **<.0001** | 8.49 | ± | 0.05 ^a^ | 7.95 | ± | 0.12 ^b^ | 7.56 | ± | 0.42 ^a, b^ | **<.0001** | 9.27 | ± | 0.04 | 8.83 | ± | 0.20 | 8.62 | ± | 0.46 | 0.0379 |
| Sugars and beverages (0-10) | 9.19 | ± | 0.04 ^a^ | 8.57 | ± | 0.06 ^b^ | 9.24 | ± | 0.21 ^a^ | **<.0001** | 9.48 | ± | 0.03 ^a^ | 8.97 | ± | 0.08 ^b^ | 9.14 | ± | 0.33 ^a, b^ | **<.0001** | 9.55 | ± | 0.03 ^a^ | 8.92 | ± | 0.22 ^b^ | 9.57 | ± | 0.20 ^a, b^ | **0.0200** |
| **Balance** |  |  |  |  |  |  |  |  |  |  |  |  |  |  |  |  |  |  |  |  |  |  |  |  |  |  |  |  |  |  |
| Carbohydrates (0-5) | 2.90 | ± | 0.04 ^a^ | 2.91 | ± | 0.04 ^b^ | 2.84 | ± | 0.21 ^a, b^ | **<.0001** | 2.45 | ± | 0.03 | 2.49 | ± | 0.07 | 2.30 | ± | 0.22 | 0.6753 | 1.63 | ± | 0.03 | 1.49 | ± | 0.15 | 2.15 | ± | 0.35 | 0.2142 |
| Fat (0-5) | 3.72 | ± | 0.04 | 3.60 | ± | 0.04 | 3.61 | ± | 0.22 | 0.0778 | 3.38 | ± | 0.03 | 3.41 | ± | 0.07 | 3.40 | ± | 0.20 | 0.9161 | 2.44 | ± | 0.04 | 2.24 | ± | 0.18 | 3.01 | ± | 0.37 | 0.1565 |
| Total energy (0-5) | 3.23 | ± | 0.04 ^a^ | 3.03 | ± | 0.05 ^b^ | 2.82 | ± | 0.26 ^a, b^ | **0.0041** | 3.29 | ± | 0.03 | 3.17 | ± | 0.07 | 3.35 | ± | 0.26 | 0.2876 | 3.30 | ± | 0.03 ^a^ | 2.41 | ± | 0.18 ^b^ | 3.47 | ± | 0.31 ^a^ | **<.0001** |
| Abbreviations: KNHANES, Korea National Health and Nutrition Examination Survey; KHEI, Korean Health Eating Index; NS, no skipping; SB, skipping breakfast; SD, skipping dinner | | | | | | | | | | | | | | | | | | | | | | | | | | | | | |  |
| 1) Continuous variables are presented as mean ± standard error. Statistical analysis used proc surveyreq for significant differences between variables. | | | | | | | | | | | | | | | | | | | | | | | | | | | | | |  |
| 2) Values in boldface are significant at *p*-value <0.05. Letters in different superscripts in the same row indicate values that are different at *p*<0.05 according to the Bon-ferroni post-hoc tests. | | | | | | | | | | | | | | | | | | | | | | | | | | | | | |  |

| **Supplemental table 2. Associations (ORs and 95% CIs) between meal-skipping patterns and the risk of hyperglycemia of adults in South Korea (KNHANES 2013-2020)** | | | | |
| --- | --- | --- | --- | --- |
|  | **Model 1** ^1)^ | **Model 2** ^2)^ | **Model 3** ^3)^ | **Model 4** ^4)^ |
|  | **OR (95% CI)** | | | |
| **Young (19 - 44y)** |  |  |  |  |
| NS | 1.000 | 1.000 | 1.000 | 1.000 |
| SB | **1.318 (1.161 - 1.496)** | **1.272 (1.118 - 1.447)** | **1.318 (1.140 - 1.525)** | **1.327 (1.142 – 1.544)** |
| SD | 0.711 (0.413 - 1.225) | 0.809 (0.468 - 1.400) | 0.959 (0.539 - 1.706) | 0.811 (0.458 – 1.435) |
| **Middle-aged (45 - 64y)** |  |  |  |  |
| NS | 1.000 | 1.000 | 1.000 | 1.000 |
| SB | **1.134 (1.015 - 1.267)** | **1.137 (1.016 - 1.274)** | **1.157 (1.021 - 1.310)** | 1.109 (0.962 - 1.251) |
| SD | 0.638 (0.439 - 0.927) | 0.780 (0.536 - 1.136) | 0.773 (0.523 - 1.140) | 0.767 (0.510 - 1.152) |
| **Elderly (≥ 65y)** |  |  |  |  |
| NS | 1.000 | 1.000 | 1.000 | 1.000 |
| SB | 0.943 (0.725 - 1.226) | 0.937 (0.719 - 1.222) | 0.979 (0.738 - 1.299) | 0.916 (0.688 - 1.220) |
| SD | **0.579 (0.353 - 0.950)** | **0.600 (0.367 - 0.983)** | **0.588 (0.360 - 0.958** | **0.487 (0.290 - 0.821)** |
| Abbreviations: OR, odds ratio; CI, confidence interval; KNHANES, Korea National Health and Nutrition Examination Survey; NS, no skipping; SB, skipping breakfast; SD, skipping dinner | | | | |
| Multivariate logistic regression analysis between the meal skipping patterns and the risk of hyperglycemia adjusted confounding factors | | | | |
| 1) Model 1 was no adjusted | | | | |
| 2) Model 2 was adjusted for sex and energy intake | | | | |
| 3) Model 3 was adjusted for sex, energy intake, marital status, household income, graduation status, exercising, residential area, smoking, drinking | | | | |
| 4) Model 4 was adjusted for sex, energy intake, marital status, household income, graduation status, exercising, residential area, smoking, drinking, BMI, family history | | | | |
